# Supplementary material for: Targeted amplification-based whole genome sequencing of Monkeypox virus in clinical specimens
Source: Microbiol Spectr. 2023 Dec 4;12(1):e02979-23. doi: 10.1128/spectrum.02979-23 (PMC10783113; doi:10.1128/spectrum.02979-23)
Supplement: Figure S1 — Maximum-likelihood tree of WGS of monkeypox virus specimens from Ontario and references with bootstrap values greater than 70 indicated at nodes. [file spectrum.02979-23-s0001.pdf]

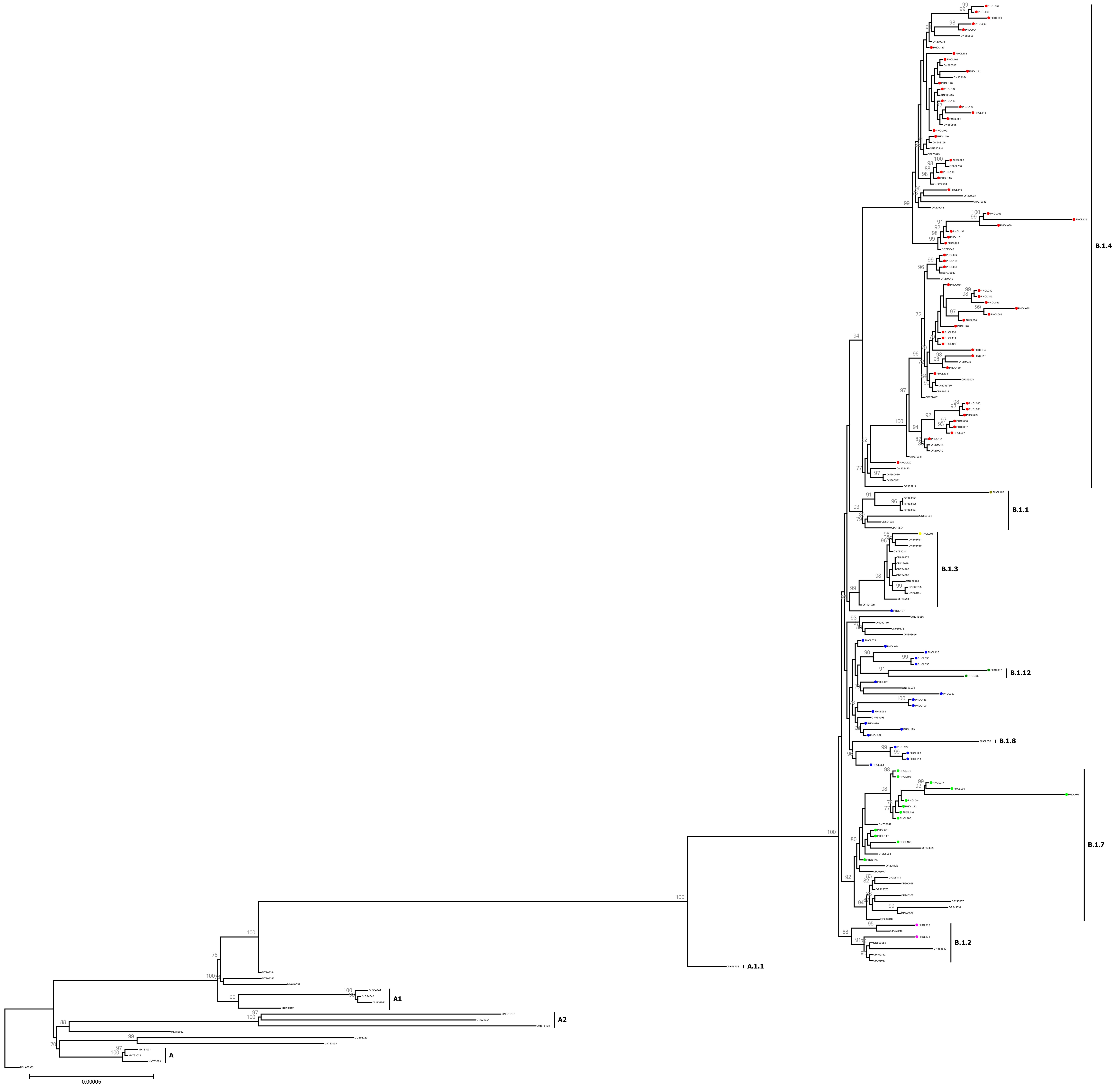

Supplementary Figure 1. Maximum-likelihood tree of WGS of Monkeypox virus specimens from Ontario and references with bootstrap values greater than 70 indicated at nodes. Phylogenetic trees were rooted using NCBI reference sequence NC_063383. A total of 195,933 positions were included in the final dataset. The scale bar represents the number of nucleotide substitutions per site. Lineages are indicated with colored circles defined above.
